# Influence of the Manner of Information Presentation on Risky Choice

**DOI:** 10.3389/fpsyg.2021.650206

**Published:** 2021-10-25

**Authors:** Hong-Zhi Liu, Zi-Han Wei, Peng Li

**Affiliations:** ^1^Computational Social Science Laboratory, Nankai University, Tianjin, China; ^2^Department of Social Psychology, Zhou Enlai School of Government, Nankai University, Tianjin, China; ^3^Key Research Base of Humanities and Social Sciences of the Ministry of Education, Academy of Psychology and Behavior, Faculty of Psychology, Tianjin Normal University, Tianjin, China; ^4^Tianjin Social Science Laboratory of Students' Mental Development and Learning, Tianjin, China; ^5^Department of Applied Social Sciences, The Hong Kong Polytechnic University, Hong Kong, Hong Kong SAR, China

**Keywords:** risky choice, information search, presentation manner, expected value maximization, attention allocation

## Abstract

We are constantly faced with decisive situations in which the options are not presented simultaneously. How the information of options is presented might influence the subsequent decision-making. For instance, presenting the information of options in an alternative- or dimension-wise manner may affect searching patterns and thus lead to different choices. In this study, the effects of this manner of information presentation on risky choice according to two experiments (Experiment 1, *N* = 45; Experiment 2, *N* = 50) are systematically examined. Specifically, two tasks with different presentation are conducted. Participants could search the information of one option (alternative-wise task) or dimension (dimension-wise task) for each time. Results revealed that the participants assigned in the alternative-wise task exhibited more choices consistent with expected value theory and took a longer decision time than those in the dimension-wise task. Moreover, the effect of task on choice was mediated by the direction of information search. These findings suggest a relationship between information search pattern and risky choice and allow for a better understanding of the mechanisms and processes involved in risky choice.

## 1. Introduction

In our daily life, we are always faced with decisive situations in which the options are not presented simultaneously. Thus, examining the effect of information presentation manner on decision-making is important. Imagine the following two scenarios:

*Scenario 1: Your investment counselor shows you two investment plans. She tells you, “In one plan, you can earn $1,000 with 90% probability. In the other plan, you can earn $1,500 with 70% probability.” Which plan will you choose?*
*Scenario 2: Your investment counselor shows you two investment plans. She tells you, “In one plan, you can earn $1,000, and in the other plan you can earn $1,500. The probabilities of earning money in the two plans are 90% and 70%.” Which plan will you choose?*

Although the information on risky options is exactly the same in the two scenarios and the decision time is unlimited, the manner of presentation may lead to different risky choices. In this study, we aim to examine the effect of presentation manner on risky choices.

In the field of decision-making under risk, mainstream theories commonly predict that for each option, individuals will weigh the value of each outcome by some function of probability, sum up all weighted values, and select the option that offers the highest overall value (Edwards, 1954; Payne and Braunstein, 1978; Basili and Chateauneuf, 2011). Prominent theories of risky choices, such as expected value (EV) theory and cumulative prospect theory (CPT) (Kahneman and Tversky, 1979; Tversky and Kahneman, 1992), all belong to the family of expectation models. For instance, EV theory assumes that individuals calculate the expected value for each option and choose the option with the highest expected value. This weighting and adding process requires the integration of all available information on the options, wherein complex computations and an *alternative-wise* information search are performed.

Other researchers proposed that people choose between risky options, relying on simplification heuristics, such as maximax heuristic and priority heuristic (Brandstätter et al., 2006). By following a heuristic process, people need not integrate information from all dimensions to reach a decision; rather, they usually rely on a single key dimension. The heuristic process requires the selective use of information on the options, wherein simple and ordinal comparisons and a *dimension-wise* information search pattern are applied. For instance, maximax heuristic assumes that individuals identify the maximum outcome of each option and choose the option with the highest monetary payoff. Empirical evidence demonstrates that different models fit certain risky tasks (Pachur et al., 2014; Barrafrem and Hausfeld, 2019; Schoemann et al., 2019), indicating that people may apply various strategies in executing different tasks.

In this study, the alternative-/dimension-wise presentation of risky information is hypothesized to influence the risky choices of individuals. Previous research showed that information search patterns correlate with the decision strategy. In risky decisions from experience, Hills and Hertwig (2010) found that individuals who switch less between options are more likely to apply the EV maximization strategy. In intertemporal choices, Reeck et al. (2017) found that manipulating the ease of dimension-wise information search patterns had a causal influence on the intertemporal choice of individuals. In their experiment, a participant moves the mouse over a relevant box to view that piece of information, and then, the information contained within that box is revealed. Researchers made either dimension- or alternative-wise transitions relatively more difficult by introducing a 1,000 ms delay between the time when a participant's cursor entered a box and the time when the information in that box was revealed. All other transitions caused the box to open immediately. The results showed that the information search of participants is affected by the manipulation, and thus, their intertemporal choices are biased. Following the same logic, in this study, presenting risky information in an alternative- or dimension-wise manner is hypothesized to manipulate the ease of information search strategies, thus promoting the choices predicted by alternative- or dimension-wise models. Therefore, we hypothesize that the risky choices of participants would be affected by the presentation manner of information.

In this study, we conducted two experiments to examine the effect of presentation manner on the risky choices of individuals. With the use of a within-subject design, the participants were asked to complete two tasks in which they could search the risky information on a desktop screen and make a choice. Given that previous work highlighted the effect of task complexity (e.g., the number of alternatives and dimensions) in determining the decision strategy adopted by individuals (Payne, 1976), we focus on the simplest type of risky options (i.e., each option contains one non-zero outcome and one corresponding probability). In the alternative-wise task, participants could press one key on the keyboard to search the information on one option and press another key to search the information on the other option. Similarly, in the dimension-wise task, information search is performed in a dimension-wise manner.

We hypothesize that participants in the alternative-wise task are more likely to adopt the alternative-wise expectation strategies, whereas participants in the dimension-wise task are more likely to adopt the dimension-wise heuristic strategies. Substantial studies have revealed that compared with the heuristic strategies, the expectation strategies elicit more choices predicted by EV theory (Rao et al., 2015; Ashby et al., 2018) and longer decision time (Su et al., 2013). Hence, the following hypothesis is posed:

H_1_: Participants in the alternative-wise task will make more EV-consistent choices and take a longer decision time than in the dimension-wise task.

We also hypothesize that the direction of information search varied between the alternative-wise and dimension-wise tasks. The participants in the alternative-wise task are prompted to adopt the alternative-wise information search, whereas those in the dimension-wise task are more likely to adopt the dimension-wise information search. Previous evidence showed that EV strategy elicits more alternative-wise information search compared with heuristic strategy (Pachur et al., 2013; Su et al., 2013). We thus infer that participants in the alternative-wise task will show more alternative-wise information search than in the dimension-wise task and thus make more EV-consistent choices. Therefore, our second hypothesis for this study is derived.

H_2_: The effect of task on EV-consistent choice will be mediated by the direction of information search.

In this study, two experiments tested the hypotheses above. In Experiment 1, we tested H_1_ by examining whether the differences in choices and decision times between the alternative-/dimension-wise tasks exist. In Experiment 2, we tested the mediation effect of the direction of information search. Data from the experiments reported in this study and Supplementary Material are publicly available *via* the Open Science Framework (https://osf.io/s29x6/).

## 2. Experiment 1

In Experiment 1, we examined the effect of presentation manner on the decision-making of individuals in simple binary gambles. In the experiment, each option contained one outcome and one corresponding probability. For the alternative-wise task, participants were instructed to press keys to search the information of one option for each time. For the dimension-wise task, participants were instructed to press keys to search the information on one dimension (i.e., outcome dimension or probability dimension) for each time.

### 2.1. Method

#### 2.1.1. Participants

Forty-five college students (*M*_*age*_ = 21.0 ± 1.8; 60% women) were recruited from a university's human subject pool to participate in this experiment. All participants had normal or corrected-to-normal vision and provided written informed consent prior to the experiment. The participants received 20 yuan (RMB; approximately US$2.9) in cash for participating and an additional amount (1–10 yuan; approximately US$0.1–$1.5) based on their performance during the experiment.

#### 2.1.2. Stimuli and Experimental Task

The stimuli consisted of 60 pairs of randomly generated risky options. All the options involved gains only, and no dominating options existed. The outcomes ranged from 1 to 99 yuan, and the probabilities ranged from 1 to 98% (see Supplementary Table 1). The probabilities were presented to the left of the outcomes. The positions of the options were counterbalanced, that is, the riskier option (gaining a greater amount with lower probability) was either on the top or the bottom. Stimuli were presented on a 17-inch LCD monitor controlled by a Dell PC with a display resolution of 1,024 × 768 pixels and a refresh rate of 60 Hz.

Two risky choice tasks were performed in this experiment, namely, alternative- and dimension-wise tasks, both of which were completed on a computer. Participants were instructed to search the information of risky options freely and choose their preferred options. In the alternative-wise task, participants were asked to search the information of one option for each time. In the dimension-wise task, participants were asked to search the information of one dimension (i.e., probability dimension or outcome dimension) each time. Each participant performed the two tasks, but performed only one task on a given day, with an interval of no <3 days between the two tasks. The order of the tasks was counterbalanced across participants. The two tasks contained the same 60 pairs of gambles. With 60 trials per condition and 45 participants, the number of data points per condition exceeded that recommended by Brysbaert and Stevens (2018).

To incentivize their cooperation further, the participants were told that one choice would be randomly selected at the end of the experiment to be treated as a real choice, with the relevant outcomes determined by a computer program. All possible outcomes (1–99 yuan) were discounted at a rate of 0.1. Therefore, the participants would receive an additional incentive (1–10 yuan) to their 20-yuan payment for participating in the experiment.

#### 2.1.3. Procedure

In each task, the participants first consented to take part in the experiment. Thereafter, they were given instructions about the experiment, and two practice trials were allowed to familiarize the participants with the task. The testing session contained 60 trials, the order of which was counterbalanced across participants. The 60 trials were divided into two blocks, with each block containing 30 trials. Participants were permitted to take a 1–2 min break after finishing each block.

**Figure 1:**
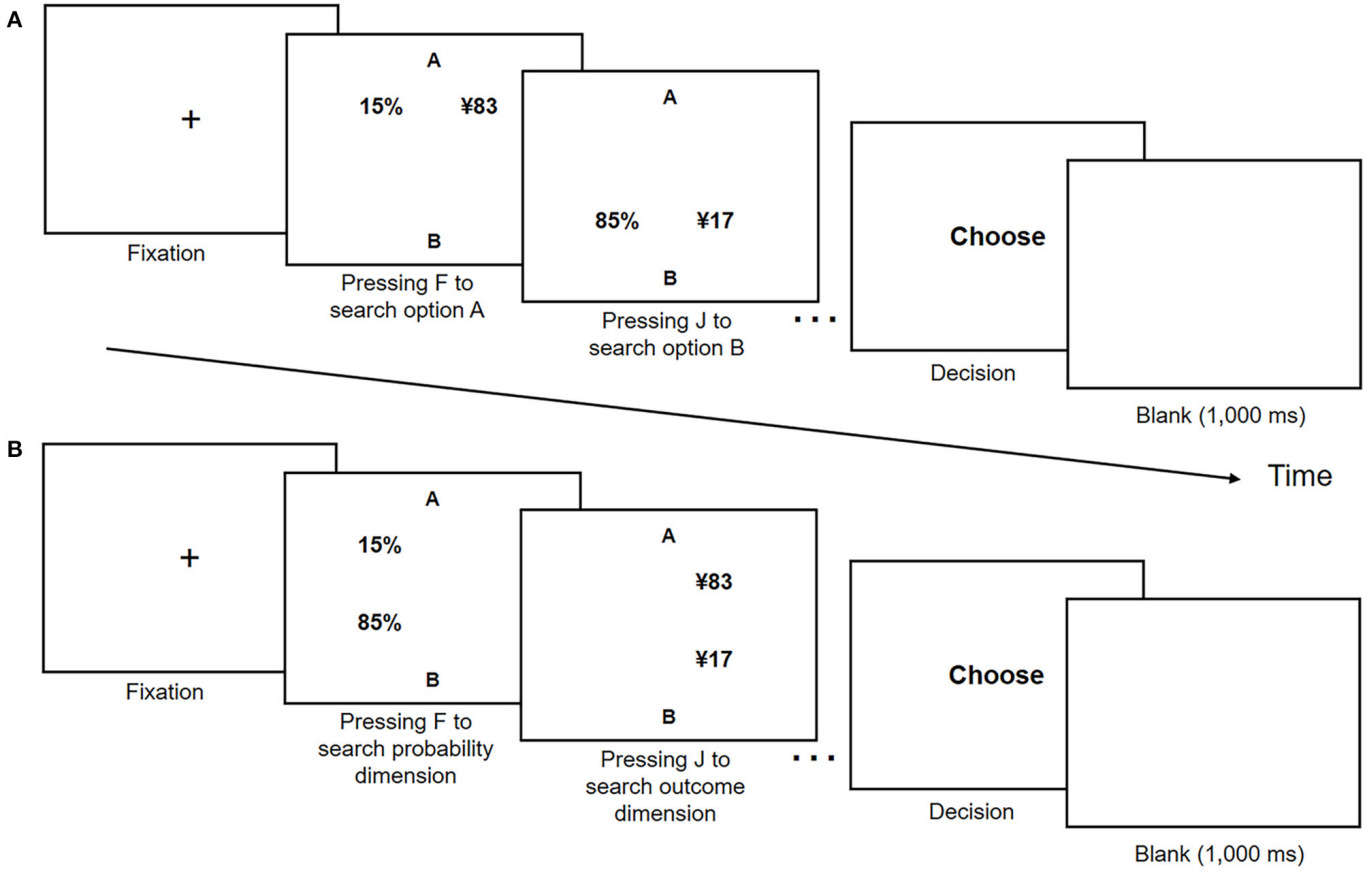
Trial procedure and timing in **(A)** alternative- and **(B)** dimension-wise tasks in Experiment 1.

At the beginning of each trial, a fixation disc was presented at the center of the display. Then, the participants were asked to press F and J on the keyboard to search the information of the risky options. In the alternative-wise task, the participants pressed F and J to search the information of options A and B, respectively. Likewise, in the dimension-wise task, the participants pressed F or J to search the information of the probability or outcome dimension, respectively. No time limit was set for searching information, and the participants were asked to press the space key to prompt the decision screen after they finished searching. Subsequently, the participants indicated their choice by pressing F (a decision for option A) or J (a decision for option B). After each participant responded, a 1,000 ms interval (with a blank screen) was shown before the next trial began. Figure 1 presents the trial procedure and timing.

#### 2.1.4. Strategy Classification

To examine the effect of presentation manner on the choices of individuals, we modeled the choices of the participants by using the EV strategy and the maximax heuristic strategy. According to the EV strategy, the weighted (by probability) outcomes of each option are integrated, and the option with the highest expected value is chosen. According to the maximax strategy, the options are compared according to their maximum outcomes, and the option with the more attractive maximum outcome is chosen. We used EV and maximax to model the choices separately for the risky choices of the participants in the two tasks. Using a maximum likelihood approach, we classified each participant to the strategy with the best fit (Pachur et al., 2014; Suter et al., 2016). Specifically, for each participant *i*, the goodness of fit of strategy *k* across *N* pairs of risky options was determined as


(1) $$\begin{array}{l} G_{i,k}^{2}=-2\overset{N}{\underset{j}{\sum }}ln\left[f_{j}\left(y\right)\right] \end{array}$$

where *f*_*j*_(*y*) represents the probability with which the strategy predicts an individual choice *y* in risky choice *j*. If option A was chosen, then *f*_*j*_(*y*) was the probability that the strategy predicted the choice of option A over option B, *p*_*j*_(A, B). If option B was chosen, then *f*_*j*_(*y*) was the probability that the strategy predicted the choice of option B, 1–*p*_*j*_(A, B). *p*_*j*_(A, B) was defined using the softmax choice rule


(2) $$\begin{array}{l} p_{j}\left(A,B\right)=\frac{e^{\varphi \cdot V\left(A\right)}}{e^{\varphi \cdot V\left(A\right)}+e^{\varphi \cdot V\left(B\right)}} \end{array}$$

where for EV, the subjective valuations of options A and B, *V*(A) and *V*(B), were defined as *V*(A) = *x*_*A*_ × *p*_*A*_ and *V*(B) = *x*_*B*_ × *p*_*B*_, respectively (with *x* and *p* being the outcome and probability of the nonzero outcomes of the option, respectively); for maximax, they were defined as *V*(A) = *x*_*A*_ and *V*(B) = *x*_*B*_. The adjustable parameter *φ* is a choice sensitivity parameter (estimated for each participant) that specifies how sensitive the predicted *p*_*j*_ is to differences in the subjective valuation of the gambles. Participants were classified as following the strategy with the best fit (i.e., lowest *G*^2^). If the best-fitting strategy *G*^2^ equalled (or was higher than) the value of *G*^2^ under random choice (i.e., with *p*[A, B] = 0.5), then the individual was classified as “guessing or using another strategy.”

#### 2.1.5. Data Analysis

We used mixed-effect models with random effects of participant and item (pairs of options) to analyze our data by using the *lme4* and *lmerTest* packages in the R statistical environment (Bates et al., 2015; Kuznetsova et al., 2017). Treating the participant and item as random factors allowed us to generalize our findings beyond specific participants and items in this study (Baayen et al., 2008; Judd et al., 2012).

### 2.2. Results

#### 2.2.1. Choices

We found that the participants made the same choice in the two tasks in 78% of the cases. To examine the effect of the task on individuals' EV maximization strategy, we conducted a mixed-effect logistic regression that predicted the EV-consistent choice by task, including the random effects for participant and item. We found that the task was a significant factor in predicting an increased likelihood of EV-consistent choice, *b* = 0.28, CI_95%_ = [0.14, 0.42], OR = 1.32, CI_95%_ = [1.15, 1.52], *z* = 3.84, *p* < 0.001. The results indicated that the dimension-wise presentation manner made participants less likely to adopt the EV maximization strategy, thus supporting H_1_.

#### 2.2.2. Strategy Classification

**Figure 2:**
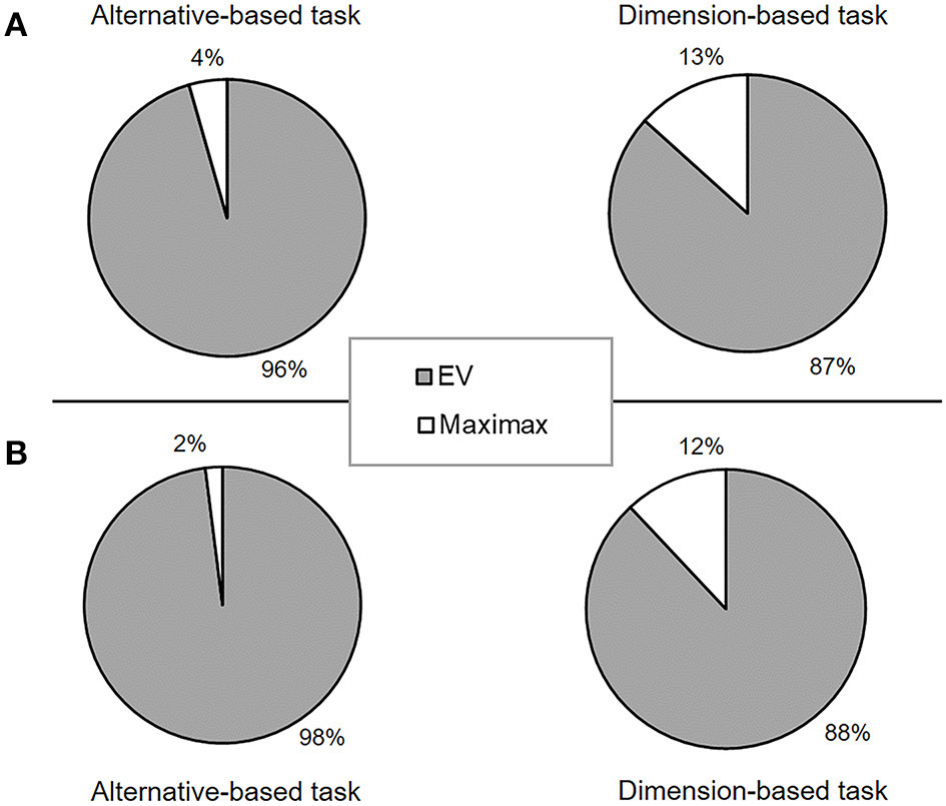
Proportion of participants classified as following the EV strategy and the maximax heuristic strategy, classified separately for the alternative-wise and the dimension-wise tasks in **(A)** Experiment 1 and **(B)** Experiment 2, respectively.

We modeled the participant choices by using the EV strategy and the maximax heuristic strategy. The best-fitting parameter values and the respective model fits of the strategies are reported in Supplementary Table 3. The distribution of participants classified as EV or maximax strategy is shown in Figure 2A. Although more participants were classified as following EV in the alternative-wise task (96%) than in the dimension-wise task (87%), the difference did not reach significance level: *z* = 1.48, *p* = 0.069.

#### 2.2.3. Decision Time

Response times (the period from searching the information until the decision prompt and then log-transformed) were examined with a mixed-effect linear regression, including the fixed effects of task (1 = alternative-wise; 0 = dimension-wise), the EV difference (the absolute value of the difference between the expected values of two options), the outcome difference (the absolute value of the difference between the outcomes of two options), and the random effects of participant and item. We found that the decision time in the alternative-wise task (*M* = 4.88 s, CI_95%_ = [4.72, 5.04]) was longer than that in the dimension-wise task (*M* = 4.13 s, CI_95%_ = [4.01, 4.25]), *b* = 0.16, CI_95%_ = [0.14, 0.19], *t* = 11.86, *p* < 0.001. The EV difference was a significant predictor that predicted the decision time, *b* = −0.006, CI_95%_ = [−0.007, −0.004], *t* = −7.32, *p* < 0.001. The outcome difference cannot significantly predict the decision time, *b* = −0.00, CI_95%_ = [−0.001, 0.001], *t* = −0.73, *p* = 0.472.

## 3. Experiment 2

In Experiment 1, we found that the presentation manner affects the simple risky choice of the individuals. In Experiment 2, we used eye-tracking technology to further test the mediation effect of the direction of information search.

### 3.1. Method

#### 3.1.1. Participants

We calculated the sample size based on the result of EV-consistent choice in Experiment 1 by using *lmmpower* function for *longpower* package in R (Donohue and Edland, 2016), with a power of 0.95 and an α error probability of 0.05. The results indicated that 2,703 samples were needed, suggesting that approximately 45 participants were needed for this experiment. Fifty college students (*M*_*age*_ = 23.9 ± 3.6; 42% women) participated in the current experiment. All participants had normal or corrected-to-normal vision and provided written informed consent prior to the experiment. The participants received 20 yuan in cash for participating, and an additional amount (1–10 yuan) based on their performance during the experiment.

#### 3.1.2. Apparatus

The eye movements of the participants were recorded by using the EyeLink 1000 Plus desk-mounted eye tracker (SR Research, Ontario, Canada) with the eye position sampled at 1,000 Hz. The visual display was presented on a 17-inch LCD monitor (with a refresh rate of 60 Hz) controlled by a Dell PC. The screen resolution was 1,024 × 768 pixels. A chin rest was used to minimize head movements and to maintain the distance between the eyes and monitor at 58 cm. When viewed from this distance, the screen subtended a visual angle of 36° horizontally and 29° vertically. Participants viewed the stimuli with both eyes, but eye movement data were collected from the right eye only. Participants responded during the experiment by pressing keys on a keyboard.

#### 3.1.3. Stimuli and Experimental Task

The stimuli were composed of 60 pairs of gambles, which were generated randomly by a computer. Different from Experiment 1, all the pairs of gambles were selected such that the maximax heuristic and EV strategy predicted opposite choices. The position of the options was counterbalanced. The values of each option (i.e., outcomes and probabilities) were presented in Arial font at a 1.3° visual angle. The (horizontal/vertical) center-to-center distance between any two values was greater than 5°, which ensured that the values were fixated properly and prevented peripheral identification of an adjacent value during fixation (Rayner, 1998, 2009).

Similar to Experiment 1, two tasks were performed in this experiment: alternative- and dimension-wise tasks.

#### 3.1.4. Procedure

After giving their consent, the participants were informed about the experiment and given a brief description of the apparatus. A five-point calibration and validation procedure was used. The maximum error of validation was 0.5° in the visual angle. After the initial calibration, two practice trials were conducted to allow the participants to familiarize themselves with the task. The testing session contained 60 trials, the order of which was counterbalanced across participants. The 60 trials were divided into two blocks, with each block containing 30 trials. Participants were permitted to take a 1–2 min break after finishing each block.

**Figure 3:**
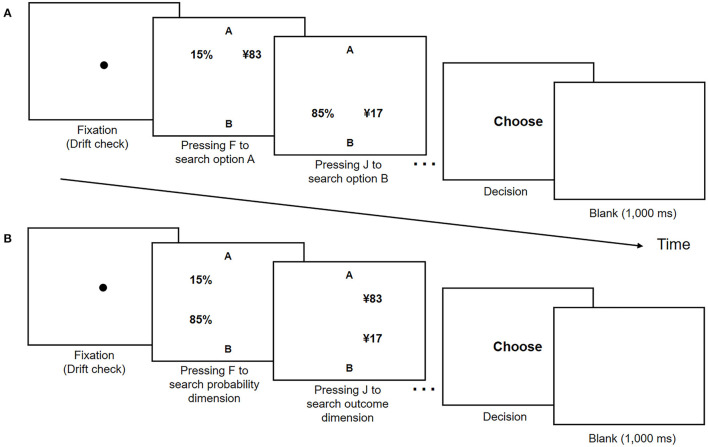
Trial procedure and timing in **(A)** alternative- and **(B)** dimension-wise tasks in Experiment 2. Each trial began with each participant fixing their gaze at the middle of the screen. After registering their response, these participants were shown a blank screen at a 1,000 ms interval before proceeding to the next trial.

At the beginning of each trial, a fixation disc was presented at the center of the display. This disc also served as a drift check for the eye tracker. When fixation on that disc was registered, the participants were asked to press F and J on the keyboard to search the information of the risky options. In the alternative-wise task, the participants pressed F and J to search the information of options A and B, respectively. Likewise, in the dimension-wise task, the participants pressed F or J to search the information of the probability or outcome dimension, respectively. No time limit was set for searching information, and the participants were asked to press the space key to prompt the decision screen after they finished searching. Subsequently, the participants indicated their choice by pressing F (a decision for option A) or J (a decision for option B). After each participant responded, a 1,000 ms interval (with a blank screen) was shown before the next trial began. Figure 3 presents the trial procedure and timing.

#### 3.1.5. Pre-processing of the Eye-Tracking Data

The collected eye movement data were analyzed by using EyeLink Data Viewer (SR Research, Ontario, Canada). Four non-overlapping, identically sized (16.2 × 11.5° visual angle) rectangular regions of interest around each piece of information (i.e., the outcomes and probabilities) were defined. Fixations were described as periods of a relatively stable gaze between two saccades, and fixations shorter than 50 ms were excluded from the analyses.

#### 3.1.6. Search Measure Index

To evaluate the overall search direction of information acquisition, we employed the search measure (SM) index proposed by Böckenholt and Hynan (1994) to combine the transition percentages into an aggregate measure


(3) $$\begin{array}{l} SM=\frac{\sqrt{N}\left[\frac{AD}{N}\left(r_{a}-r_{d}\right)-\left(D-A\right)\right]}{\sqrt{A^{2}\left(D-1\right)+D^{2}\left(A-1\right)}} \end{array}$$

where *A* and *D* denote the number of options and the number of dimensions, respectively (i.e., in this experiment, *A* = 2, *D* = 2); *r*_*a*_ and *r*_*d*_ denote the number of alternative-wise transitions and dimension-wise transitions, respectively, and *N* denotes the number of total transitions. The predominance of alternative-wise transitions increases with an increasing value of SM index (Su et al., 2013). A negative value of SM index indicates a predominantly dimension-wise search, and a positive value indicates a predominantly alternative-wise search (Pachur et al., 2013).

### 3.2. Results

#### 3.2.1. Choices

We found that in 82% of the cases, the participants made the same choice in the two tasks. To examine the effect of the task on individuals' EV maximization strategy, we performed a mixed-effect logistic regression that predicted the EV-consistent choice by task, including the random effects of participant and item. We found that task was a significant factor that predicted an increased likelihood of EV-consistent choice, *b* = 0.73, CI_95%_ = [0.56, 0.90], OR = 2.08, CI_95%_ = [1.76, 2.45], *z* = 8.59, *p* < 0.001. The results of EV-consistent choice indicated that the dimension-wise search manner made people less likely to adopt the EV maximization strategy. Thus, H_1_ was supported.

#### 3.2.2. Strategy Classification

Similar to Experiment 1, we classified each participant to the strategy with the best fit. The distribution of participants classified as EV or maximax strategy is shown in Figure 2B. The results revealed that more participants were classified as following the EV strategy in the alternative-wise task (98%) than in the dimension-wise task (88%), *z* = 1.96, *p* = 0.025. The results suggest that the difference between the alternative-wise task and dimension-wise task may, at least in part, be attributed to people's use of different strategies.

#### 3.2.3. Decision Time

Similar to Experiment 1, decision times were examined with a mixed-effect linear regression, including the fixed effects of task, EV difference, outcome difference, and the random effects of participant and item. We found that the decision time in the alternative-wise task (*M* = 4.32 s, CI_95%_ = [4.17, 4.48]) was longer than that in the dimension-wise task (*M* = 3.86 s, CI_95%_ = [3.76, 3.96]), *b* = 0.08, CI_95%_ = [0.05, 0.10], *t* = 6.31, *p* < 0.001. The EV difference was a significant predictor that predicted the decision time, *b* = −0.009, CI_95%_ = [−0.011, −0.007], *t* = −8.06, *p* < 0.001. The outcome difference cannot significantly predict the decision time, *b* = −0.00, CI_95%_ = [−0.002, 0.001], *t* = −0.44, *p* = 0.665.

#### 3.2.4. Search Measure Index

We found that the SM index in the alternative-wise task (*M* = 1.87, CI_95%_ = [1.83, 1.91]) was significantly greater than that in the dimension-wise task (*M* = −0.98, CI_95%_ = [−1.01, −0.94]), *b* = 2.85, CI_95%_ = [2.80, 2.90], *t* = 109.03, *p* < 0.001.

To test whether the effect of task on EV-consistent choice was mediated by the SM index, we used the GAMLj module (Gallucci, 2019) in jamovi (jamovi project, 2019) to perform mediation analysis. We hypothesized that compared to the dimension-wise task, the alternative-wise task increased the alternative-wise information search, thereby leading to more EV-consistent choices.

The task (independent variable) was entered as a dummy-coded variable (1 = the alternative-wise task; 0 = the dimension-wise task), EV-consistent choice (dependent variable) was entered as a dummy-coded variable (1 = choose the option with higher EV; 0 = choose the option with lower EV), and the SM index was the mediator. The numbers of participants and items were controlled as covariates. We generated 95% CI on the basis of 5,000 bootstrap samples.

**Figure 4:**
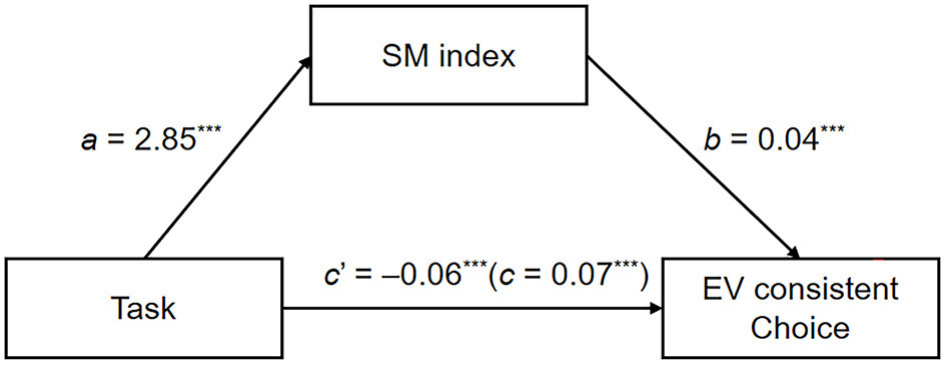
Results of the mediation analysis of the SM index in Experiment 2. The indirect effect is the product of coefficients *a* and *b*. The coefficients in parentheses are the total effect (i.e., sum of indirect and direct effects). ^***^p < 0.001.

Figure 4 shows the results of the mediation analysis through the SM index. The total and direct effects of task on the EV-consistent choice were *c* = 0.07, CI_95%_ = [0.05, 0.09], *z* = 7.15, *p* < 0.001 and *c*' = −0.06, CI_95%_ = [−0.09, −0.02], *z* = −3.50, *p* < 0.001, respectively, and the total indirect effect through the SM index (mediator) was *ab* = 0.12, CI_95%_ = [0.10, 0.15], *z* = 10.04, and *p* < 0.001. The results of mediation analysis supported H_2_.

## 4. Discussion and Conclusion

In this study, we conducted two experiments to systematically examine the effects of the manner of information presentation on simple binary gambles (Experiment 1) and further examine the mediation effect of direction of information search (Experiment 2). The results revealed that (1) compared with the participants in the dimension-wise task, those who performed the alternative-wise task were more likely to adopt the EV maximization strategy; (2) the decision time in the alternative-wise task was greater than that in the dimension-wise task; and (3) participants in the alternative-wise task showed more alternative-wise information search and thus exhibited more EV-consistent choices than in the dimension-wise task.

Our findings indicate that the decision strategies during risky choices can be affected by the presentation manner. Specifically, the participants showed more EV-consistent choices and required a longer decision time in the alternative-wise task than in the dimension-wise task in both experiments. EV theory usually assumes a complex computation process and predicts a longer decision time (Su et al., 2013). Therefore, the results suggest that individuals may use multiple strategies in risky choices and shift between these strategies as a function of task and strategic variability, which is consistent with previous studies (Venkatraman et al., 2009; Ashby et al., 2018; Popovic et al., 2019).

We found that most of the participants were classified as following the EV strategy (92% in Experiment 1 and 93% in Experiment 2). One reason for this result might be that we focused on the simple binary gambles (i.e., between pairs of options, each consisting of a probability *p* to win amount *x*) in this study. In this condition, the participants had sufficient cognitive resources to execute the EV maximization calculation. This condition can also explain why we found that the EV difference can significantly predict the decision time but the outcome difference cannot. Previous research that used simple binary gambles also found that the alternative-wise process models decisively outperformed dimension-wise ones in accounting for choices and decision times (Glickman et al., 2019), which is consistent with our findings.

A detail that is worth noting is that although the expectation models can be interpreted as describing strategies that adopt the weighting and adding process (Pachur et al., 2013; Su et al., 2013), someone may argue that the interpretation of expectation models as process models may sometimes be overly simplistic. However, a recent work revealed that the parameters of CPT can reflect selective attention allocation (Pachur et al., 2018), indicating that the as-if model can also reflect the characteristics of information processing. Glöckner and Betsch (2008) also argued that the weighting and adding process can be accomplished by the intuitive system and provided process evidence.

The findings of this study have implications on the cognitive process during risky decision-making. We found that the alternative-/dimension-wise tasks influenced participants' direction of information search, thus leading to different choices. The alternative-wise task promotes alternative-wise comparisons and thus enhances the possibility of adopting the EV maximization strategy. Similarly, the dimension-wise task promotes dimension-wise comparisons and hampers the possibility of using the EV maximization strategy. Substantial research has shown that the risky choices of the individuals in different tasks are always accompanied by a varied SM index value (Pachur et al., 2013, 2014; Su et al., 2013; Pfeiffer et al., 2014), which reflects the direction of information search. The finding that the SM index mediated the effect of task on choices indicates that the direction of transitions plays an important role in the process during risky choice.

The above results add to the wealth of evidence that supports the causal link between information process and risky decision-making. Previous studies focused on the perspective of attention allocation and revealed that both alternative-wise and dimension-wise relative attention are associated with subsequent risky choice (Fiedler and Glöckner, 2012; Pachur et al., 2013; Brandstätter and Körner, 2014; Stewart et al., 2015). Sui et al. (2020) found that the manipulations on both alternative-wise and dimension-wise relative attention can have a causal influence on risky choices. This study revealed that the manipulation on the ease of strategies can also influence the risky choice of the individuals, providing a new perspective to examine the causal link. Future studies may consider developing a new paradigm to manipulate the strategies in a straightforward manner to bias the risky choice of the individuals.

Our results have implications for risky decision-making in real-world contexts. The findings, which indicated that risky decisions can be affected by a convenient manipulation of presentation manner, suggests a potential application of an intervention improving an individual decision-making by using a similar presentation set. Looking back at the aforementioned scenarios, if the investment counselor expects you to make a choice wise on expectation theories, then she should present the information in an alternative-wise manner to help in your decision-making.

We acknowledge some constraints in this study. First, the probabilities were always presented to the left of the outcomes in both experiments. Given that decision-makers generally prefer to read from left to right (Orquin and Loose, 2013), the unbalanced position of outcomes/probabilities on the left/right may lead to more attention on the probabilities. Future studies may consider presenting the gambles in an ellipsoid display format (Glöckner and Herbold, 2011) to eliminate this confounding effect. Second, this study used a within-subject design and did not employ a control condition. Future studies are encouraged to include a control task, for example, a task in which the information of options can be shown on the screen simultaneously, thus enabling the effect of representation manner to be evaluated exactly.

In conclusion, this study indicates that the manipulation on the manner of information presentation can systematically influence the subsequent risky choices.

## Data Availability Statement

The datasets presented in this study can be found in online repositories. The names of the repository/repositories and accession number(s) can be found in the article/Supplementary Material.

## Ethics Statement

The studies involving human participants were reviewed and approved by Institutional Review Board of Psychology of Nankai University. The patients/participants provided their written informed consent to participate in this study.

## Author Contributions

H-ZL and Z-HW conceived and designed this study and wrote the paper. H-ZL, PL, and Z-HW designed experimental stimuli and procedures. PL implemented experimental protocols and collected data. H-ZL and PL analyzed data. All authors contributed to the article and approved the submitted version.

## Funding

This work was partially supported by the National Natural Science Foundation of China (Nos. 72001158, 71901126), the Humanity and Social Science Youth Foundation of Ministry of Education of China (No. 19YJC190013), and the Fundamental Research Funds for the Central Universities (No. 63212065).

## Conflict of Interest

The authors declare that the research was conducted in the absence of any commercial or financial relationships that could be construed as a potential conflict of interest.

## Publisher's Note

All claims expressed in this article are solely those of the authors and do not necessarily represent those of their affiliated organizations, or those of the publisher, the editors and the reviewers. Any product that may be evaluated in this article, or claim that may be made by its manufacturer, is not guaranteed or endorsed by the publisher.
