# Supplementary material for: Influence of the Manner of Information Presentation on Risky Choice
Source: Front Psychol. 2021 Oct 25;12:650206. doi: 10.3389/fpsyg.2021.650206 (PMC8573322; doi:10.3389/fpsyg.2021.650206)
Supplement: Supplementary file 1 [file Data_Sheet_1.PDF]

Supplemental Material for

Influence of the manner of information presentation on risky choice

Table S1

Experimental Stimuli in Experiment 1

Item	Alternative A		Alternative B	
	Outcome	Probability	Outcome	Probability
1	¥46	8%	¥8	53%
2	¥41	5%	¥10	31%
3	¥10	44%	¥96	4%
4	¥57	63%	¥91	32%
5	¥77	8%	¥30	35%
6	¥28	75%	¥62	24%
7	¥7	86%	¥81	3%
8	¥72	3%	¥9	54%
9	¥92	7%	¥18	77%
10	¥63	75%	¥85	55%
11	¥9	55%	¥78	3%
12	¥39	19%	¥14	62%
13	¥18	4%	¥5	33%
14	¥14	25%	¥4	92%
15	¥57	17%	¥18	65%
16	¥17	88%	¥82	14%
17	¥59	24%	¥21	82%
18	¥49	96%	¥68	75%
19	¥82	40%	¥63	53%
20	¥25	69%	¥86	11%
21	¥80	16%	¥34	46%
22	¥68	32%	¥30	82%
23	¥50	51%	¥96	25%
24	¥65	22%	¥38	47%
25	¥99	19%	¥22	90%
26	¥98	29%	¥51	73%
27	¥35	20%	¥20	45%
28	¥16	73%	¥34	29%
29	¥82	32%	¥40	67%
30	¥70	66%	¥69	67%
31	¥41	34%	¥67	3%
32	¥58	58%	¥34	92%
33	¥59	33%	¥26	45%
34	¥2	70%	¥7	6%
35	¥37	91%	¥42	11%
36	¥74	67%	¥79	23%
37	¥58	91%	¥97	48%
38	¥79	36%	¥64	38%

39	¥1	24%	¥86	19%
40	¥73	71%	¥17	75%
41	¥21	83%	¥65	11%
42	¥77	2%	¥12	92%
43	¥39	52%	¥53	45%
44	¥78	85%	¥94	12%
45	¥22	98%	¥92	97%
46	¥97	79%	¥6	95%
47	¥56	28%	¥14	85%
48	¥36	60%	¥44	6%
49	¥60	18%	¥88	17%
50	¥38	98%	¥74	32%
51	¥17	70%	¥42	6%
52	¥44	97%	¥59	90%
53	¥40	53%	¥45	14%
54	¥38	19%	¥7	94%
55	¥30	44%	¥92	41%
56	¥48	61%	¥88	1%
57	¥46	22%	¥36	46%
58	¥73	7%	¥70	84%
59	¥63	12%	¥27	82%
60	¥41	90%	¥88	18%

Table S2

Experimental Stimuli in Experiment 2

Item	Alternative A		Alternative B	
	Outcome	Probability	Outcome	Probability
1	¥53	16%	¥19	45%
2	¥80	10%	¥63	35%
3	¥46	5%	¥38	88%
4	¥96	42%	¥90	65%
5	¥95	4%	¥68	56%
6	¥78	9%	¥15	87%
7	¥90	16%	¥35	61%
8	¥96	44%	¥63	74%
9	¥94	8%	¥13	94%
10	¥51	21%	¥26	53%
11	¥77	43%	¥50	85%
12	¥89	1%	¥18	96%
13	¥34	49%	¥17	99%
14	¥41	32%	¥28	61%
15	¥88	8%	¥49	56%
16	¥26	24%	¥23	59%
17	¥33	23%	¥24	58%
18	¥90	29%	¥75	73%
19	¥74	6%	¥51	14%
20	¥88	34%	¥77	76%
21	¥61	34%	¥30	91%
22	¥85	35%	¥79	85%
23	¥70	37%	¥42	92%
24	¥89	13%	¥79	83%
25	¥88	10%	¥51	44%
26	¥47	35%	¥39	91%
27	¥71	5%	¥26	56%
28	¥73	28%	¥56	74%
29	¥56	1%	¥47	57%
30	¥73	1%	¥37	56%
31	¥96	10%	¥82	58%
32	¥49	15%	¥39	74%
33	¥64	1%	¥22	75%
34	¥74	24%	¥57	59%
35	¥86	8%	¥77	20%
36	¥70	3%	¥50	96%
37	¥55	18%	¥48	70%
38	¥95	34%	¥57	58%

39	¥58	30%	¥25	83%
40	¥72	43%	¥64	54%
41	¥89	4%	¥40	82%
42	¥94	35%	¥70	94%
43	¥78	64%	¥51	98%
44	¥79	21%	¥56	71%
45	¥61	50%	¥59	83%
46	¥69	12%	¥40	79%
47	¥38	10%	¥22	43%
48	¥94	3%	¥67	15%
49	¥68	52%	¥66	93%
50	¥17	20%	¥10	40%
51	¥94	23%	¥84	89%
52	¥63	10%	¥18	37%
53	¥19	4%	¥15	52%
54	¥96	33%	¥87	51%
55	¥94	65%	¥88	83%
56	¥96	30%	¥62	63%
57	¥57	14%	¥55	66%
58	¥60	10%	¥40	44%
59	¥56	28%	¥35	72%
60	¥87	44%	¥71	68%

Table S3

Best-fitting Parameter Values for Strategy Classification in Experiment 1 and Experiment 2

		Alternative-based task		Dimension-based task	
		EV	Maximax	EV	Maximax
Experiment 1	φ (*M*)	0.218	0.005	0.220	0.007
	G^2 (*M*)	56.82	80.43	58.62	79.47
Experiment 2	φ (*M*)	0.699	0.001	0.362	0.004
	G^2 (*M*)	35.02	83.01	44.74	82.15

Note. G^2 indicates the goodness of fit. Shown are the best-fitting values of the choice sensitivity parameter and goodness of fit of the EV strategy and maximax strategy when modeling each participant's choices in the two tasks.
